# PEDOT:PSS Interfaces Support the Development of Neuronal Synaptic Networks with Reduced Neuroglia Response *In vitro*

**DOI:** 10.3389/fnins.2015.00521

**Published:** 2016-01-14

**Authors:** Giada Cellot, Paola Lagonegro, Giuseppe Tarabella, Denis Scaini, Filippo Fabbri, Salvatore Iannotta, Maurizio Prato, Giancarlo Salviati, Laura Ballerini

**Affiliations:** ^1^Department of Neuroscience, International School for Advanced StudiesTrieste, Italy; ^2^IMEM-CNR IstitutoParma, Italy; ^3^ELETTRA Synchrotron Light SourceTrieste, Italy; ^4^Department of Life Science, University of TriesteTrieste, Italy; ^5^Department of Chemical and Pharmaceutical Sciences, University of TriesteTrieste, Italy

**Keywords:** PEDOT:PSS, hippocampal neurons, glial cells, neural interfaces, conductive polymers, single cell patch clamp

## Abstract

The design of electrodes based on conductive polymers in brain-machine interface technology offers the opportunity to exploit variably manufactured materials to reduce gliosis, indeed the most common brain response to chronically implanted neural electrodes. In fact, the use of conductive polymers, finely tailored in their physical-chemical properties, might result in electrodes with improved adaptability to the brain tissue and increased charge-transfer efficiency. Here we interfaced poly(3,4-ethylenedioxythiophene):poly(styrene sulfonate) (PEDOT:PSS) doped with different amounts of ethylene glycol (EG) with rat hippocampal primary cultures grown for 3 weeks on these synthetic substrates. We used immunofluorescence and scanning electron microscopy (SEM) combined to single cell electrophysiology to assess the biocompatibility of PEDOT:PSS in terms of neuronal growth and synapse formation. We investigated neuronal morphology, density and electrical activity. We reported the novel observation that opposite to neurons, glial cell density was progressively reduced, hinting at the ability of this material to down regulate glial reaction. Thus, PEDOT:PSS is an attractive candidate for the design of new implantable electrodes, controlling the extent of glial reactivity without affecting neuronal viability and function.

## Introduction

Brain-machines interfaces are prosthetic devices designed to ameliorate the prognosis of neurological patients by targeting non-specific sensory/motor deficits in brain injuries, or more specific symptoms in Parkinson's disease, chronic pain and epilepsy (Donoghue, 2002; Machado et al., 2010; Goodrick, 2014).

Recording/stimulation electrodes are the core component of implanted interfaces and need to show excellent and stable electrical properties together with a good biocompatibility once chronically exposed to the biological environment.

The most common cause of implant failure is the brain tissue reaction against the electrodes (Polikov et al., 2005). This reaction favors the glial-scar formation leading to devices with high impedance and poor electrical charge transfer (Lempka et al., 2009).

The physical and chemical properties of conducting polymers (CPs) allow the development of devices characterized by improved adaptation to the brain tissue (Green et al., 2008; Williamson et al., 2015). Together with adaptability, CP electrodes are characterized by low impedance when interfaced to excitable tissues. Both these features improve the quality of the recorded signals (Ludwig et al., 2006) and are required in the design of high-performing electrodes.

CPs is composed by repeating units of covalently bonded monomers organized in highly conductive films when polymerized in the presence of anionic dopants (Heeger, 2001). By varying the production process and the type of dopant it is possible to tune CPs mechanical and electrical performance (Dimitrieva et al., 2009). Within CPs, poly(3,4-ethylenedioxythiophene):poly(styrene sulfonate) (PEDOT:PSS) is a suitable candidate for the development of high-performance electrode-coatings toward biomedical applications (Schmidt et al., 1997; Cui et al., 2003; Ludwig et al., 2006; Xiao et al., 2006; Berggren and Richter-Dahlfors, 2007; Richardson-Burns et al., 2007; Collazos-Castro et al., 2010; Forcelli et al., 2012). This is motivated by PEDOT:PSS chemical stability in aqueous environment (Yamato et al., 1995) combined to the ease at which PEDOT:PSS physical features can be tuned. For instance, when PEDOT:PSS is doped with substances of decreasing molecular size, the surface roughness of CP films increases (Baek et al., 2014). A similar effect results from increasing the concentrations of the PSS in the electro polymerization solution (Tamburri et al., 2009).

Primary embryonic neurons were shown to survive once embedded in PEDOT:PSS, (Richardson-Burns et al., 2007) and the long-term adhesion of neuronal cells on PEDOT:PSS substrates was improved by pre-layering the CP surface with poly-lysine (Collazos-Castro et al., 2010).

PEDOT:PSS has been polymerized to coat multi-electrode arrays (MEAs). In these experiments the stimulation delivered by PEDOT:PSS layered electrodes evoked stronger neuronal responses when compared to uncoated ones (Nyberg et al., 2007).

It has been recently shown that adaptable electrode arrays, realized on a thin film of parylene-C and containing microelectrodes made by PEDOT:PSS can be used for *in vivo* electrocorticography (ECoG) in rats. In these experiments sharp-wave events mimicking epileptic spikes were successfully recorded (Khodagholy et al., 2011). In a further development, electrochemical transistor (OECT) based on PEDOT:PSS, used to record brain activity *in vivo*, displayed a superior signal-to-noise ratio (SNR) when compared with surface electrodes (Khodagholy et al., 2013).

In this technology area, one of the more advanced results is represented by the NeuroGrid: an organic material–based, ultra-conformable, biocompatible, and scalable neural interface array with neuron-sized-density electrodes, exploited for *in vivo* recording of superficial cortical activity (Khodagholy et al., 2015).

Notwithstanding these studies, the impact of PEDOT:PSS interfaces on neuronal synaptic activity and glial-cell reactivity has not been fully investigated. This is a relevant issue, in particular when CPs electrodes are involved in the design of long-term implants (Lempka et al., 2009; Gunasekera et al., 2015).

In the present study we investigated the biocompatibility of PEDOT:PSS layers when challenged with postnatal brain cells (neurons and neuroglia) to optimize their use in future prosthetic devices. The electrical and morphological properties of PEDOT:PSS doped with different concentrations of ethylene glycol (EG) were analyzed. To assess the viability and performance of neurons and synaptic networks we developed short- and long-term hippocampal cultures (i.e., 1 or 3 weeks *in vitro*) and we performed single cell electrophysiology, scanning electron microscopy (SEM) and immunofluorescence to document synaptic activity, cell morphology and density when interfaced to PEDOT:PSS films. We investigated glial reactivity to PEDOT:PSS substrates by immunofluorescence technique.

## Materials and methods

### Preparation of PEDOT:PSS layers

Poly(3,4-ethylenedioxythiophene):poly(styrenesulfonate), termed PEDOT:PSS, type Clevios PH1000 was purchased from Heraeus Conductive Polymers Division.

Successively PEDOT:PSS solution was firstly doped with a 0.05 vol. of dodecyl benzene sulfonic acid (DBSA) surfactant (Sigma Aldrich) for efficient film forming, and then different concentrations v/v (0, 1, and 3%) of EG were added to enhance electrical conductivity with respect to the pristine state (Crispin et al., 2003; Ouyang et al., 2004; Romeo et al., 2015). One solution was prepared without EG addition and used as reference control.

Homogeneous PEDOT:PSS films at different EG concentrations were spin coated on a glass slides of 1 × 3 cm at 1500 rpm for 30 s. The final films thickness was around 80 nm, as measured with the profilometer (Tarabella et al., 2013). Slides were finally baked on a hot plate at 140 C for 60 min.

### Electrical characterization of PEDOT:PSS layers

Electrical measurements of PEDOT:PSS layer were obtained by 2-point source/measure precision unit (Agilent B2902A) and controlled by homemade LabView software. The I-V characteristics are acquired, after the realization of Silver Ohmic contacts distant 1 cm, spanning the applied voltage from −1 V to +1 V achieved in 0.1 V steps and recording the resulting current flowing in the PEDOT:PSS layer. Different measurements were realized with different EG doping levels of PEDOT:PSS.

### Contact angle

Five micro liter of water were dropped on the surfaces and the images captured using a homemade static contact angle measuring system. The equilibrium contact angle was evaluated by a statistical study of several images (Chen et al., 2005): two layers where investigated for each EG concentration and the measurement were repeated three times for each sample.

### Atomic force microscopy (AFM)

AFM was used for studying at high-resolution the three-dimensional reconstructions of PEDOT:PSS layers at three different EG doping conditions (0, 1, and 3%). All AFM images were acquired using a commercially available microscope (Solver Pro AFM from NT-MDT—NT-MDT Co.—Moscow—Russia) endowed with a closed-loop scanner. Measurements were carried out in air at room temperature working in dynamic mode. Cantilevers, characterized by a resonant frequency of 90 kHz and a force constant of 1.74 nN/nm (NSG03 series from NT-MDT—NT-MDT Co.—Moscow—Russia) were used working at low oscillation amplitudes with half free-amplitude set point. High-resolution images were 512 × 512 pixels frames acquired at 1 lines/s scan speed working in air. All AFM data were analyzed using Gwyddion (Nečas and Klapetek, 2013), scanning probe microscopy data analysis free software. Surface roughness was computed as the root-mean-square (RMS) value of the height irregularities of AFM images. The ratio between the surface area and the projected surface area, S_*r*_, was computed from AFM images by simple triangulation and correlated to roughness values.

### Ethical approval

All experiments were performed in accordance with the European Community Council Directive of 24 November 1986 (86/609EEC) and Italian law (decree 26/14) and were approved by local Authority Veterinary Service.

The University of Trieste Animal Facility (Life Sciences Department, Italy, authorized by the Italian Ministry of Health) hosted animals and breeding conditions and procedures complied with the 2010/63/UE EU guidelines and Italian law (26/14).

Neonatal rats were sacrificed by rapid decapitation and the tissue of interest (hippocampus) harvested, all efforts were made to minimize suffering. The work was performed on explanted tissue and did not require ethical approval.

### Cultures and immunofluorescence

Hippocampal neurons were isolated from postnatal (P2-3) rat pups and seeded with a standard amount of cells (~150,000 cells/coverslips) as previously reported (Lovat et al., 2005) on different substrates. Cells were plated on glass (control cultures) or PEDOT:PSS (with different concentrations of EG, pretreated with plasma cleaning) coverslips, both previously layered with poly-ornithine (Sigma-Aldrich), to increase permissiveness of surfaces. Each coverslip was incubated with 300 μl of poly-ornithine containing solution for a least 1 h, then the drop was removed and cells seeded. Cultured cells were used for experiments at 8–22 days *in vitro (*DIV*)*.

To quantify neuronal and glial cells density, cultures (control and PEDOT:PSS) were immune-labeled following the procedure previously described (Cellot et al., 2011). Briefly, cultures were fixed with 4% paraformaldehyde in PBS (20 min), then, upon washout in PBS, incubated in blocking solution and subsequently incubated with rabbit polyclonal antibody against β tubulin III (1:250 dilution; Sigma-Aldrich) and mouse monoclonal antibody against GFAP (1:200 dilution; Sigma-Aldrich). Upon washout in PBS, cultures were incubated with the secondary goat anti-rabbit Alexa Fluor 594 (1:500, Invitrogen) and goat anti-mouse Alexa Fluor 488 (1:500, Invitrogen). Two culture series (4 fields for each slide) were sampled for each condition. Samples were visualized with a Leica DM 6000 microscope at 20x magnification. Offline analysis of the images was performed with the open source image-processing package Fiji.

The content of GFAP was estimated by selecting squared areas (25 μm^2^) close to the nucleus in randomly chosen glial cells. The mean intensity of fluorescence within this region was calculated using dedicated tool of Fiji software. Background fluorescence, measured in region of sample without cells, was subtracted from fluorescence values (Salazar et al., 2008). Area of single glial cells, whose perimeter was manually drawn, was automatically measured by the software in randomly selected cells, sufficiently isolated from neighbors.

### Scanning electron microscopy (SEM)

SEM was used to investigate the morphology of cellular cultures developed on PEDOT:PSS substrates and on controls. Images were acquired collecting secondary electrons on a Gemini SUPRA 40SEM (Carl Zeiss NTS GmbH, Oberkochen, Germany) working at an acceleration voltage of 5 keV. Before SEM characterization cellular samples were washed with 0.1 M cacodylate buffer (pH = 7.2) and fixed with a solution containing 2% glutaraldehyde (Fluka, Italy) in 0.1 M cacodylate buffer for 1 h at RT. Cultures were then washed in a cacodylate buffer and dehydrated by dipping in water/ethanol solutions at progressively higher alcohol concentrations (50, 75, 90, 95, 98, and 100% ethanol for 10 min each). Afterwards samples were let in 100% ethanol to dry at 4°C overnight. Prior to SEM imaging samples were gold metalized in a metal sputter coater (Polaron SC7620). We explore the neuronal morphology in terms of number of neuritis exiting the cell soma. In doing this we sampled in all groups individual neurons clearly isolated from other cells. When investigating the mean number of neuritis departing from the cell soma (Lovat et al., 2005) in these samples we found no significant differences between control and PEDOT:PSS substrates (control: 3 ± 0.2, PEDOT 0%EG: 3.4 ± 0.3, PEDOT 1%EG: 2.9 ± 0.4 neurites/cell, *n* = 8 for each condition).

### Electrophysiology

A patch-clamp amplifier (multiclamp 700b, Axon Instruments, Sunnyvale, CA, USA) was used to record visually identified (with an upright microscope equipped with differential interference contrast optics and infrared video camera) neurons, using the patch-clamp technique in voltage and current modes. Whole-cell recordings were obtained with pipettes (4–7 MΩ, Hingelberg, Malsfeld, Germany) containing 120 mM K gluconate, 20 mM KCl, 10 mM HEPES, 10 mM EGTA, 2 mM MgCl_2_, and 2 mM Na_2_ATP (pH 7.35 adding KOH). The external solution contained the following: 150 mm NaCl, 4 mm KCl, 1 mm MgCl_2_, 2 mm CaCl_2_, 10 mm HEPES, 10 mm glucose, pH 7.4. All experiments were performed at 18–22°C.

Liquid junction potential was 13 mV; membrane potential values were not corrected for it. All recordings were performed at −58 mV of holding potential. Cells exhibiting > 15% changes in either series resistance or holding were excluded from the analysis. The series resistance was < 20 MΩ and it was not compensated.

### Data analysis

Data were transferred to a computer hard disk after digitization with an A/D converter (Digidata 1322, Molecular Devices). Data acquisition (digitized at 10 kHz and filtered at 2 kHz) was performed with pClamp 9.2 software (Molecular Devices, Sunnyvale, CA, USA).

In voltage clamp mode, cells were stimulated with a 100 ms lasting hyperpolarizing stimulus (10 mV), then, in the recording, area below the capacitative transients was measured and normalized for voltage transient amplitude to calculate cellular capacitance; input resistance was obtained through Ohm's law, by measuring the amplitude of steady state current generated by voltage transient. Spontaneous postsynaptic currents were analyzed using pClamp 9 (Molecular Devices, Sunnyvale, CA, USA). This program uses a detection algorithm based on a sliding template. The template did not induce any bias in the sampling of events because it was moved along the data trace by one point at a time and was optimally scaled to fit the data at each position. All the collected events were averaged and the amplitude of current was calculated as that of the mean trace.

Statistical significance was tested using unpaired Student *t*-test (Origin, Northampton, MA, USA). A *p* < 0.05 was considered as statistically significant. Values are given as mean ± SEM.

## Results

### Physical and morphological analysis of PEDOT:PSS

PEDOT:PSS layers doped with different amounts of EG were characterized for their physical and morphological properties before using them as growth substrates for culturing hippocampal cells.

**Figure 1:**
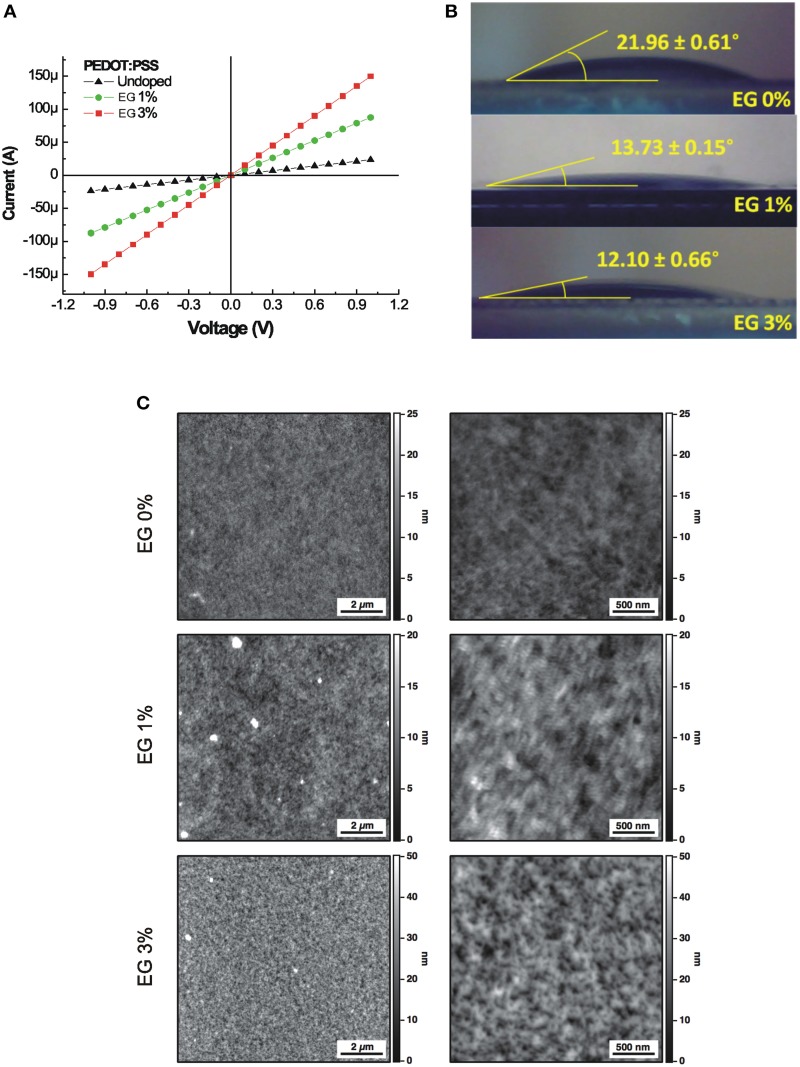
**(A)** Current-Voltage characteristics of the PEDOT:PSS layers doped with different concentration of EG. **(B)** Water contact angles of the PEDOT:PSS layers doped with different concentration of EG. **(C)** 10 × 10 μm^2^ AFM images (left column) and the corresponding 2.5 × 2.5 μm^2^ magnifications (right column) of PEDOT:PSS layers doped with increasing amounts of EG (0, 1, and 3%). Brighter color areas in the image correspond to higher portions of the sample.

The I–V characteristics of PEDOT:PSS layer doped with different concentration of EG, varying from undoped (0%) up to the 3% are shown in Figure 1A. The electrical conductance shows an increase of about one order of magnitude, increasing the concentration of EG. In fact, undoped PEDOT:PSS layer shows an electrical conductance of 2.35 × 10^−5^ S, meanwhile the electrical conductance increases up to 1.5 × 10^−4^ S in the case of the 3% doped films.

The digital images of water equilibrium contact angle of PEDOT:PSS layers doped with different concentration of EG are reported in Figure 1B. The equilibrium contact angle decreases from 21.96° to 12.10° with increasing EG from 0 to 3%. The static contact angle analysis reveals that the wettability of the PEDOT:PSS layer increases with the same concentrations of EG used to improve conductance.

AFM measures on 10 × 10 μm^2^ sampled areas show a relatively uniform surface for all samples while, at the nanoscale, surfaces display different granularity (Figure 1C). Roughness analysis reveals values of 1.73, 2.38, and 6.69 nm for PEDOT:PSS doped with 0, 1, and 3% EG, respectively. Obtained roughness values are slightly higher than values characterizing cleaned glass substrates (Henke et al., 2002) and similar polymeric materials used for cell development (e.g., polydimethylsiloxane, PDMS; Palchesko et al., 2012). For a comprehensive description of PEDOT:PSS surface characteristics refer to Kim et al. (2011).

Surface analysis highlighted values of the ratio (S_r_) between the effective surface area and the projected surface area of 1.0059, 1.0018, and 1.030 for the three substrates. These values correspond to an increase of the exposed surface of about 0.6, 0.2, and 3%, respectively for 0, 1, and 3% EG contents, compared to a perfectly flat surface characterized by a unitary value of S_r_.

### PEDOT:PSS layers supporting the growth of hippocampal cultures

Upon characterization the EG doped PEDOT:PSS layers were used as growth substrates for hippocampal cultures. Neuronal and glial cells were analyzed at different times of their *in vitro* development.

#### Undoped PEDOT:PSS and PEDOT:PSS 1% EG are permissive substrates for neuronal cultures

In a first set of experiments, we investigated whether the EG dopant addition in PEDOT:PSS layers affected short-term hippocampal cultures viability.

**Figure 2:**
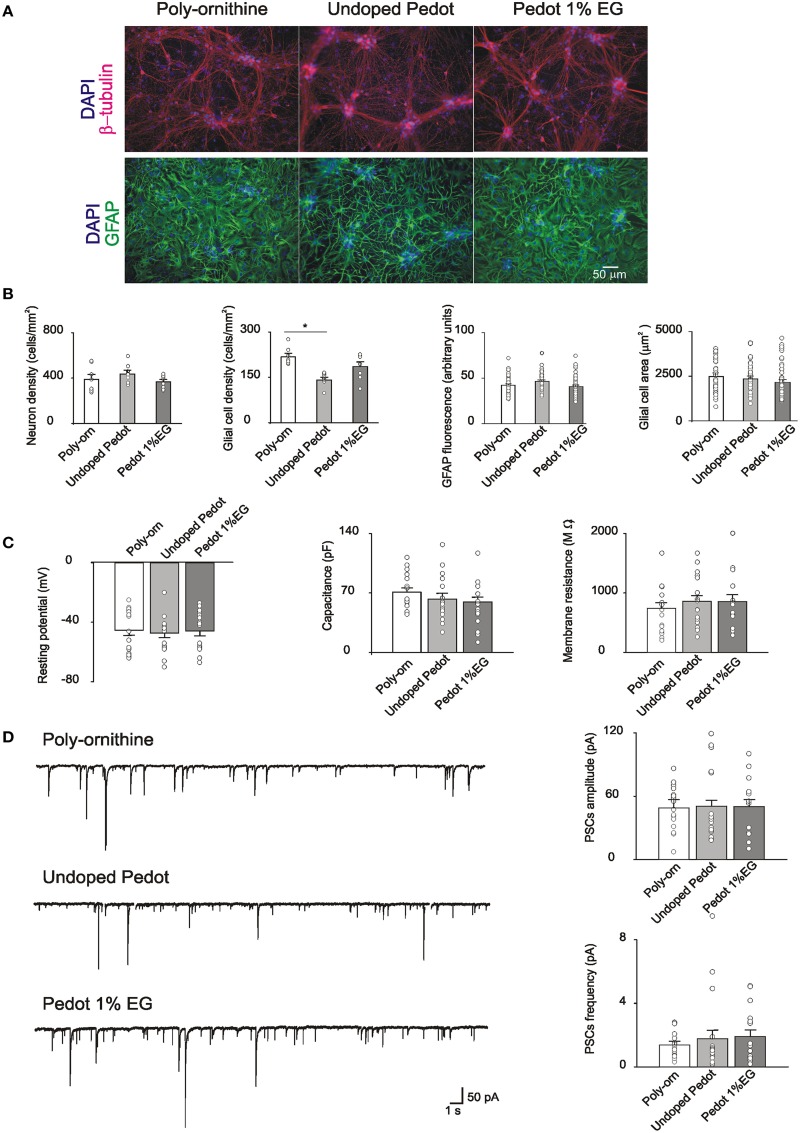
**(A)** Immunofluorescence images of cultures grown on poly-ornithine, undoped PEDOT:PSS (0% EG) and PEDOT:PSS 1% EG. Cells were stained with antibodies anti-β-tubulin (red), GFAP (green) and nuclei were marked with DAPI (blue) **(B)**. Bar plots show mean values of neuronal and glial cells density, GFAP fluorescence intensity and glial cells areas in the different conditions of growth. Superimposed dots to the bars are values from single fields (for cell density) or from single cells (for GFAP intensity and glial cell areas). Note that the glial cell density is significantly decreased on undoped PEDOT:PSS respect to the poly-ornithine control (^*^p < 0.05, *T*-test). **(C)** Bar plots are mean values of resting membrane potentials, capacitance and membrane resistance. Superimposed dots to the bars are values from single experiments. **(D)** On the left, exemplificative recordings from neurons grown on poly-ornithine, undoped PEDOT:PSS and PEDOT:PSS 1% EG respectively. On the right, bar plots show the means values of spontaneous PSCs amplitude and frequency in the different conditions of growth. Superimposed dots to the bar are values from single experiments.

Neonatal rat hippocampal cells were seeded on undoped PEDOT:PSS (0% EG), PEDOT:PSS 1% EG and control substrates. Cells attached to all the substrates and began extending neurites within the first 24 h. The first set of experiments was performed after 8 DIV, a time known to allow for synaptic network development *in vitro* (Lovat et al., 2005; Mazzatenta et al., 2007; Cellot et al., 2011). Cultures grown on PEDOT:PSS and on control substrates were fixed for immunofluorescence to report cell culture composition and relative cell density. β-tubulin III-positive neurons and GFAP–positive glial cells (Fabbro et al., 2012) are shown in Figure 2A. Neuronal cell density is similar between control and PEDOT:PSS layers (388 ± 40, 434 ± 29, and 366 ± 20 cells/mm^2^, respectively control, undoped PEDOT:PSS and PEDOT:PSS 1% EG, *n* = 8 fields each). On the contrary, glial cell density appears reduced on PEDOT:PSS layers with respect to the control (217 ± 10 cells/mm^2^ in control, 140 ± 8 cells/mm^2^ on undoped PEDOT:PSS and 185 ± 15 cells/mm^2^ on PEDOT:PSS 1% EG), although the difference was statistically significant only between undoped PEDOT:PSS and control (*p* < 0.005, *T*-test; Figure 2B).

We further quantify the intensity of GFAP fluorescence and the dimension (area size) of single glial cells in the different culture groups. No differences were observed in the measured parameters (GFAP fluorescence: control 42 ± 1.4 arbitrary units, undoped PEDOT 46 ± 1.7 arbitrary units and PEDOT 1% EG 40 ± 1.7 arbitrary units; glial cells area: control 2483 ± 202 μm^2^, undoped PEDOT 2345 ± 168 μm^2^ and PEDOT 1% EG 2143 ± 165 μm^2^, *n* = 43, *n* = 40, *n* = 44 respectively; Figure 2B).

The viability of neurons grown on PEDOT:PSS layers was assessed by single cell patch clamp recordings. Neuronal passive properties, such as the resting membrane potential, membrane capacitance and input resistance, were measured. These parameters are commonly considered as indicators of neuronal health (Carp, 1992; Djuric et al., 2015; Gao et al., 2015).

Neurons grown on PEDOT:PSS layers did not show any statistically significant differences with respect to the control in these values (resting membrane potential: −45 ± 4, −47 ± 3, and −46 ± 3 mV; capacitance: 71 ± 19, 63 ± 26, and 59 ± 6 pF; input resistance: 740 ± 98, 857 ± 98, 855 ± 119 MΩ, respectively control *n* = 16, undoped PEDOT:PSS *n* = 18, PEDOT:PSS 1% EG *n* = 15; Figure 2C).

Neurons grown on PEDOT:PSS layers showed spontaneous synaptic activity made up by a mixed population of postsynaptic currents (detected as inward currents in our recording conditions; Figure 2D; Cellot et al., 2011) indicating that such substrates support the growth of neurons connected by functionally active synapses. sPSCs amplitude and frequency on PEDOT:PSS did not differ from control ones (sPSCs amplitude: 50 ± 5, 49 ± 8, and 50 ± 7 pA; sPSCs frequency: 1.4 ± 0.2, 1.8 ± 0.5, and 1.9 ± 0.4 Hz for control *n* = 17, undoped PEDOT:PSS *n* = 18 and PEDOT:PSS 1% EG *n* = 16; Figure 2D). These results further support the similar size of control and PEDOT:PSS neuronal networks after 1 week of *in vitro* growth.

#### Long term hippocampal cultures on 1% EG and 3% EG PEDOT:PSS

PEDOT:PSS containing EG has higher electrical conductivity than EG free one (Wang et al., 2005; Nardes et al., 2008), thus, in view of the potential of this material for prosthetic applications, we tested neurons seeded on PEDOT:PSS 1 and 3% EG, to verify the viability of long term cultures on PEDOT:PSS layers.

**Figure 3:**
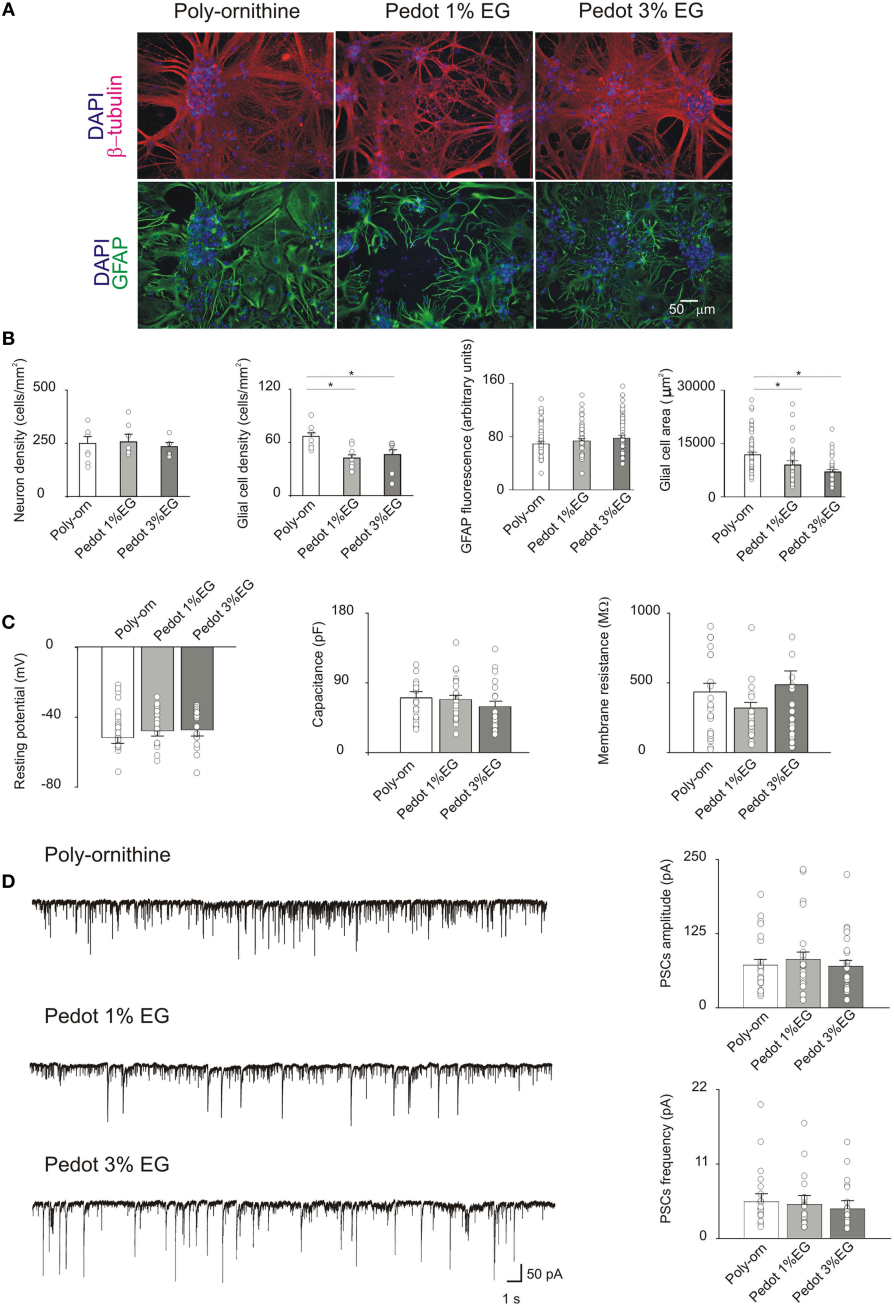
**(A)** Immunofluorescence images of cultures grown on poly-ornithine, PEDOT:PSS 1% EG and PEDOT:PSS 3% EG. Cells were stained with antibodies anti-β-tubulin (red), GFAP (green) and nuclei were marked with DAPI (blue) **(B)**. Bar plots show mean values of neuronal and glial cells density, GFAP fluorescence intensity and glial cells areas in the different conditions of growth. Superimposed dots to the bars are values from single fields (for cell density) or from single cells (for GFAP intensity and glial cell areas). Note that the glial cell density and area are significantly decreased on both PEDOT:PSS 1% EG and 3% EG respect to the poly-ornithine control (^*^p < 0.05, *T*-test). **(C)** Bar plots are mean values of resting membrane potentials, capacitance and membrane resistance. Superimposed dots to the bars are values from single experiments. **(D)** On the left, exemplificative recordings from neurons grown on poly-ornithine, PEDOT:PSS 1% EG and PEDOT:PSS 3% EG respectively. On the right, bar plots show the means values of spontaneous PSCs amplitude and frequency in the different conditions of growth. Superimposed dots to the bar are values from single experiments.

After 21 DIV (Figure 3A) neuronal density did not differ between the PEDOT:PSS and controls (249 ± 33, 256 ± 34, and 234 ± 20 cells/mm^2^, respectively for control, PEDOT:PSS 1% EG and PEDOT:PSS 3% EG, *n* = 8 fields each). Conversely, in PEDOT:PSS GFAP-positive glial cells were significantly reduced upon 3 weeks of culturing (65 ± 4 cells/mm^2^ in control, 42 ± 4 cells/mm^2^ on PEDOT:PSS 1% EG and 45 ± 6 cells/mm^2^ on PEDOT:PSS 3% EG; *p* < 0.05, *T*-Test, Figure 3B).

In these samples GFAP fluorescence intensity was found to be similar among the various conditions of growth (GFAP fluorescence: control 69 ± 3.2 arbitrary units, PEDOT:PSS 1% EG 73 ± 3.7 arbitrary units and PEDOT:PSS 3% EG 78 ± 4 arbitrary units); however GFAP-positive cell area values in PEDOT:PSS in PEDOT:PSS were significantly smaller with respect to controls (glial cells area: control 11,882 ± 758 μm^2^, PEDOT:PSS 1% EG 9018 ± 1209 μm^2^ and PEDOT:PSS 3% EG 7053 ± 633 μm^2^, *n* = 56, *n* = 37, *n* = 37, respectively; *p* < 0.05, *T*-Test, Figure 3B).

Similar to what detected in 8 DIV cultures, the membrane passive properties of sampled neurons did not differ among the three groups showing on average a resting membrane potential of −48 ± 2, −46 ± 2, and −45 ± 2 mV; capacitance values of 70 ± 8, 68 ± 6, and 59 ± 6 pF and input resistance values of 414 ± 55, 302 ± 34, 464 ± 90 MΩ, in control *n* = 25, PEDOT:PSS 1% EG *n* = 25, PEDOT:PSS 3% EG *n* = 24, respectively (Figure 3C).

In the very same conditions, 21 DIV, the measured sPSC frequency and amplitude were unaltered in PEDOT:PSS layers in respect to age matched controls (sPSCs amplitude: 71 ± 9, 81 ± 12, and 69 ± 10 pA; sPSCs frequency: 5.6 ± 1.3, 5.3 ± 1.4, and 4.6 ± 1.3 Hz for control *n* = 25, PEDOT:PSS 1% EG *n* = 25 and PEDOT:PSS 3% EG *n* = 24; Figure 3D).

#### SEM images show healthy morphology of neurons grown on PEDOT:PSS layers

**Figure 4:**
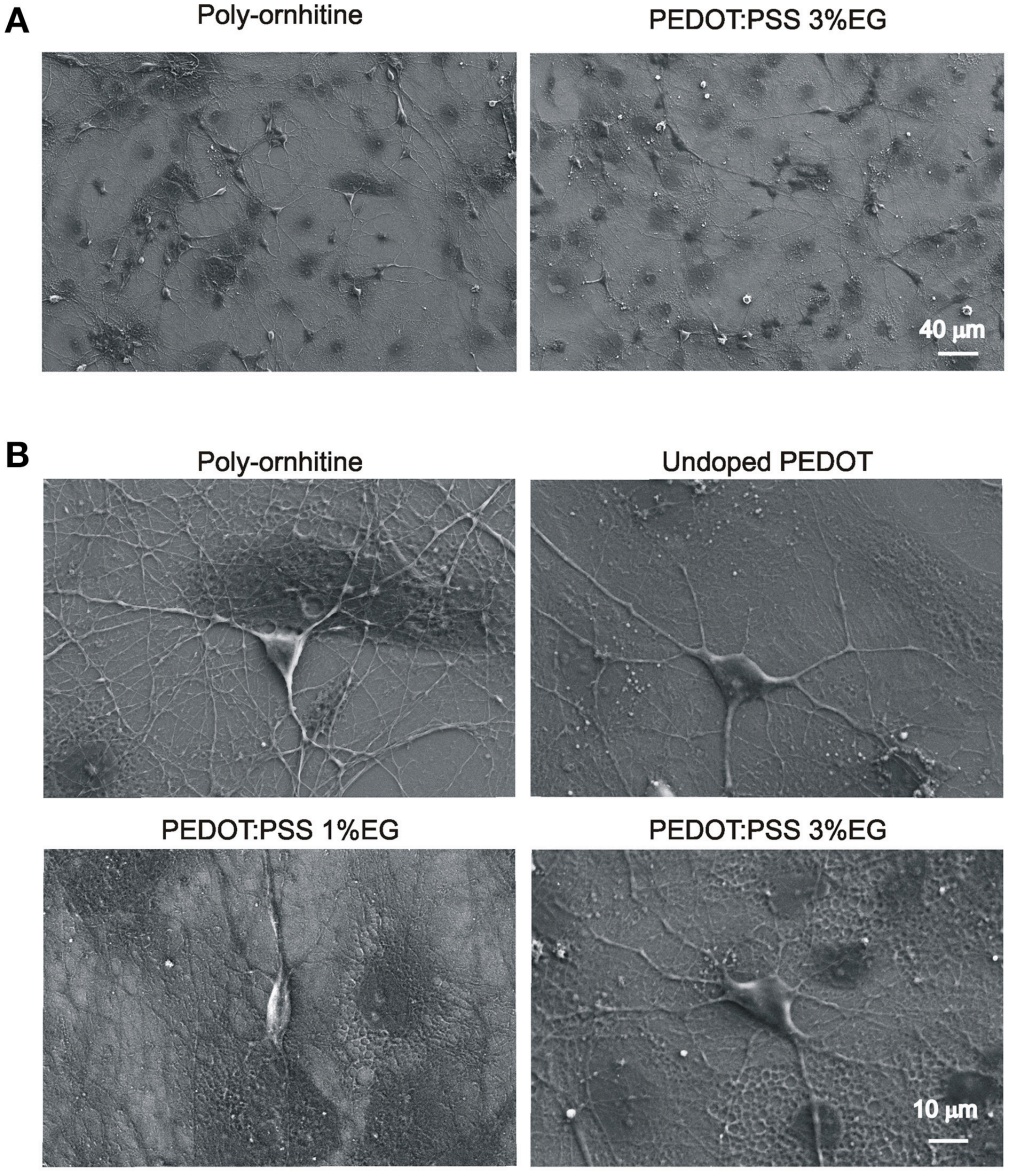
**Scanning electron microscopy micrographs showing hippocampal cultures grown on PEDOT:PSS layers. (A)** Lower magnification micrographs display that qualitatively the size of neuronal network is comparable between undoped PEDOT:PSS and PEDOT:PSS 3% EG. **(B)** Higher magnification micrographs show the healthy morphology of single neurons grown on the different substrates.

Figure 4A shows neuronal network visualized by low magnification SEM microscopy. Darker areas correspond to glial cells. Higher magnification of micrographs (Figure 4B) shows the healthy appearance of single neurons when developed on PEDOT:PSS layers, similar to that of controls. In all the conditions of growth, 3–4 neurites (see methods) emerged from the soma and extend centrifugally on the substrates, organized in intricate arborizations.

## Discussion

We studied PEDOT:PSS growth substrates in long-term hippocampal cultures, in the perspective of exploiting such materials for neural prosthesis. Implantable electrodes technology is continuously evolving also in terms of the chemistry of the material used, to improve the device longevity and performance. In particular, highly conductive materials should be design also to minimize tissue damage and reduce glial reactions (Lempka et al., 2009). In this framework, we tested primary hippocampal cells cultured interfaced to PEDOT:PSS doped with different concentrations of EG (0, 1, and 3%), a dopant reported to increase the electrical properties of conductive polymers (Crispin et al., 2003; Ouyang et al., 2004, 2015; Romeo et al., 2015). We describe here for the first time that EG not only affects the conductivity of the layers, but also improves wettability and roughness, and selectively reduces glial cell reaction. Importantly, neuronal maturation, synapse formation and function are unaltered by these substrates.

We confirmed the linear relationship between the conductive properties of PEDOT:PSS and the amount of EG dopant. In addition, the analysis of the static contact angle on the PEDOT:PSS layers indicated the ability of EG in controlling the hydrophilicity of the surface. The electrical conductivity and the wettability of the PEDOT:PSS layers are two fundamental properties for developing long-term implants, in fact high electrical conductivity allows an improved transport of the neural electrical signals and the higher wettability makes the PEDOT:PSS surfaces more hydrophilic and therefore suitable for the adhesion of living cells.

The roughness analysis of PEDOT:PSS layers showed an increase in surface RMS values as the amount of EG was increased. Compared to the control 0% EG, PEDOT:PSS 1% EG shows an increase in roughness of about 37% while PEDOT:PSS 3% EG presents a nearly three times higher roughness (287%). S_r_ ratio between the effective surface area and the projected area presents a small decrease in PEDOT:PSS 1% EG samples than 0% EG controls, but PEDOT:PSS 3% shows a valuable increase of the exposed area (about 3% larger than controls, see Results). This may potentially lead to improved charge exchange efficacy in the PEDOT:PSS 3% EG.

The biocompatibility of PEDOT:PSS was tested by culturing dissociated postnatal hippocampal cells isolated from neonatal rats. This is a widely adopted and characterized *in vitro* model for neuronal networks formation and for synaptic physiology studies. Neurons, after a few hours from seeding, are able to attach to the substrates, if permissive, and begin to extend neurites. In the following days, neurites elongate, take contact with other neurons, forming synapses that are functionally active after a week *in vitro*. Spontaneous synaptic activity comprises both excitatory and inhibitory postsynaptic currents as observed *in vivo* (Segal, 1983; Köller et al., 1990; Siebler et al., 1993). By SEM, we observed that neurons grown on PEDOT:PSS layers show a healthy morphology, with many neurites extending from the soma and forming arborizations, similarly to cells on control substrates. Also the size of the network was comparable, as immunofluorescence experiments show, where the density of neurons that attached and developed on PEDOT:PSS substrates was similar to that of control, both at 1 and 3 weeks *in vitro*.

We further characterized neurons developed on PEDOT:PSS from a functional point of view via electrophysiological recordings (Lovat et al., 2005; Cellot et al., 2011). Although electrical activity from neuronal populations grown on conductive polymers has been partially characterized previously by MEAs (Nyberg et al., 2007), in our study, by single cell patch clamp technique, we measured the single neuron membrane properties as well as those of single synaptic events.

Our experiments revealed that single cell membrane passive properties, known indicators of neuronal health (Carp, 1992; Djuric et al., 2015; Gao et al., 2015), and the spontaneous activity of the network, measured in terms of amplitude and frequency of sPSCs, were fully comparable between cultures grown on PEDOT:PSS and controls. This was observed for neurons after a week in culture (undoped and 1% EG), but also in long-term cultures (3 weeks *in vitro*) with even higher concentrations of dopant (up to 3% EG). Thus, on PEDOT:PSS interfaces we develop healthy neurons and functionally active synaptic networks, comparable to those grown on traditional peptide-layered substrates (controls).

Intriguingly, PEDOT:PSS down-regulated glial cells, with a partial reduction detected also after 1 week of culturing. The GFAP-positive glia shows clear reduction in cell density and glial cells have smaller GFAP-positive area after being interfaced for 3 weeks on PEDOT:PSS, with respect to control. Notably, the intensity of GFAP fluorescence signal appeared similar among all tested groups.

An increased number of glial cells and an enhanced glial cellular size are usually associated with pathological states (Yang and Wang, 2015), thus PEDOT:PSS apparently reduces glial response, a relevant feature for exploiting electrode-material able to induce low glial reactions (Lempka et al., 2009).

Other conductive polymers, such as PEDOP and P3MT, but not PEDOT, were reported to reduce astrocyte responses (Forcelli et al., 2012). However, in the PEDOT layers reported by Forcelli et al. (2012) PSS was not used as counter ion dopant and the deposition process was also different, probably accounting for the different results obtained.

It is interesting to note that the reduced glial response observed on PEDOT:PSS layers in our long-term cultures seems not to influence the functional properties of neuronal networks, as our experiments detected similar neuronal density and comparable electrical activity between PEDOT:PSS and controls.

It is possible that the neuronal network developed on PEDOT substrates in the presence of a reduced number of glial cells could keep intact its functional properties thanks to the hydrophilic nature of the material. This would allow the accumulation of extracellular matrix proteins (Van Kooten et al., 1992), creating a favorable environment for neuronal growth (Masuda-Nakagawa and Wiedemann, 1992).

Thus, our findings suggest that PEDOT:PSS materials can be exploited for the development of new implantable devices. We speculate that such materials *in vivo* can control the glial reactivity, with limited effects on neuronal viability. Certainly this hypothesis requires being validated trough further experiments.

## Author contributions

GC performed cell biology, electrophysiology and immunofluorescence experiments and analysis; PL, GT, FF, SI, MP, and GS contributed to material design, production and characterization; DS performed SEM and AFM experiments; LB and GS conceived the study; LB conceived the biological experimental design; GS, MP, and LB provided funding; GC and LB wrote the manuscript. All authors have given approval to the final version of the manuscript.

## Funding

We acknowledge financial support from the NEUROSCAFFOLDS-FP7-NMP-604263 and PRIN-MIUR n. 2012MYESZW.

### Conflict of interest statement

The authors declare that the research was conducted in the absence of any commercial or financial relationships that could be construed as a potential conflict of interest.
